# Neuronal Swelling: A Non-osmotic Consequence of Spreading Depolarization

**DOI:** 10.1007/s12028-021-01326-w

**Published:** 2021-09-08

**Authors:** Julia A. Hellas, R. David Andrew

**Affiliations:** Center for Neuroscience Studies, Queen’s University, Kingston, ON K7L 3N6 Canada

**Keywords:** Osmolar concentration, Osmolality, Ischemia, Cytotoxic cerebral edema, Brain edema, Water intoxication, Inappropriate ADH syndrome, SIADH, Stroke, Spreading depolarization

## Abstract

An acute reduction in plasma osmolality causes rapid uptake of water by astrocytes but not by neurons, whereas both cell types swell as a consequence of lost blood flow (ischemia). Either hypoosmolality or ischemia can displace the brain downwards, potentially causing death. However, these disorders are fundamentally different at the cellular level. Astrocytes osmotically swell or shrink because they express functional water channels (aquaporins), whereas neurons lack functional aquaporins and thus maintain their volume. Yet both neurons *and* astrocytes immediately swell when blood flow to the brain is compromised (cytotoxic edema) as following stroke onset, sudden cardiac arrest, or traumatic brain injury. In each situation, neuronal swelling is the *direct* result of spreading depolarization (SD) generated when the ATP-dependent sodium/potassium ATPase (the Na^+^/K^+^ pump) is compromised. The simple, and incorrect, textbook explanation for neuronal swelling is that increased Na^+^ influx passively draws Cl^−^ into the cell, with water following by osmosis via some unknown conduit. We first review the strong evidence that mammalian neurons resist volume change during acute osmotic stress. We then contrast this with their dramatic swelling during ischemia. Counter-intuitively, recent research argues that ischemic swelling of neurons is non-osmotic, involving ion/water cotransporters as well as at least one known amino acid water pump. While incompletely understood, these mechanisms argue against the dogma that neuronal swelling involves water uptake driven by an osmotic gradient with aquaporins as the conduit. Promoting clinical recovery from neuronal cytotoxic edema evoked by spreading depolarizations requires a far better understanding of molecular water pumps and ion/water cotransporters that act to rebalance water shifts during brain ischemia.

## Introduction

The brain is a unique organ in that lost blood flow to a small region can induce permanent dysfunction of the individual. Ischemic stroke involves brain vessel blockage, which can quickly cause irreversible neurological impairment or death. Stroke is now ranked as the second leading cause of death globally [1]. Within minutes, brain tissue is deprived of oxygen and glucose, halting adenosine triphosphate (ATP) production, and causing ATP-dependent failure of the sodium/potassium ATPase (the Na^+^/K^+^ pump). This evokes immediate loss of membrane potential in the form of a spreading depolarization (SD). Similarly, the ischemia arising from sudden cardiac arrest or from traumatic brain injury (TBI) also elicits SD. This combined metabolic stress of ischemia and SD quiets neurons but elicits significant brain cell swelling. SD can recur in the less-stressed penumbra, expanding injury but also providing a therapeutic window to reduce SD-related injury. Acute brain swelling is dangerous and often deadly because it downwardly displaces the brainstem. The mass effect damages brain tissue as it shifts the brain across the falx cerebri, tentorium cerebelli, or foramen magnum. Despite its severity, the mechanisms underlying acute cerebral swelling are poorly-characterized at the cellular level.

This review will reexamine the currently accepted processes thought to underly brain cell swelling. We will differentiate between osmotic and ischemic cell swelling, focusing on acute swelling (lasting seconds to minutes), but also considering subacute swelling (hours to days). Finally, several potential mechanisms will be reviewed which underly neuronal water accumulation during acute ischemia, and mechanisms mediating brain volume reduction and recovery. By no means are these processes well defined yet. Here, we review primarily brain slice and intact animal imaging studies that have monitored cell volume changes over seconds to minutes. We do not review isolated cell studies because these often use highly unphysiological osmotic shifts that the intact brain cannot survive [2, 3].

The pathology of cerebral edema is categorized as cytotoxic edema, involving intracellular water build-up over minutes and hours. Additionally, vasogenic edema involves accumulated extracellular fluid resulting from blood–brain barrier (BBB) disruption and serum protein extravasation over hours and days [4]. As fluid and osmolytes enter the brain’s extracellular space (ECS) from leaky vessels, vasogenic edema exacerbates swelling, thereby worsening intracerebral compression, which may lead to irreversible damage to vital brainstem structures [5]. That pathology is separate from changes to brain cell excitability, which is altered by swelling and is in hypoosmotic versus ischemic situations*.* This review will focus on cytotoxic edema, the common cause of neurological damage in cases of ischemia [5]; however, both types of swelling are dangerous and potentially fatal.

Altering osmolality while observing cellular brain swelling in situ is difficult [3, 6, 7], so the particular mechanisms underlying intracellular water buildup in neurons have commonly been studied in live brain slices where osmotic shifts can be mediated quickly, or where ischemia can be simulated using oxygen–glucose deprivation (OGD).

## Brain Cell Swelling with Osmotic Shifts

**Fig. 1:**
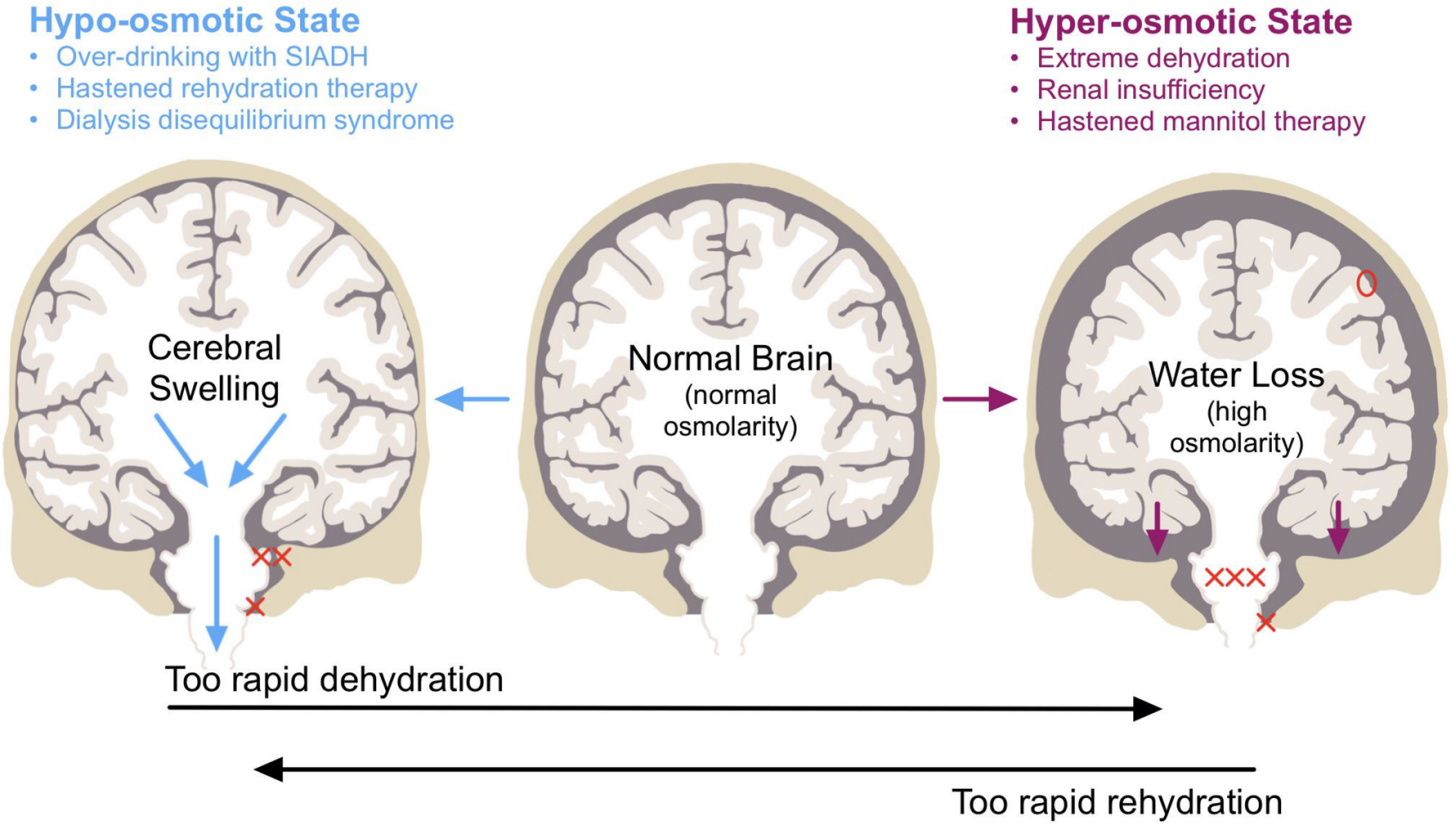
Shifts in plasma osmolality over tens of minutes can lead to brain injury. A sudden drop in plasma osmolality (left panel) leads to brain swelling as astrocytes in particular swell. Seizure is a common result as the brain becomes more excitable. In extreme cases, downward displacement of the brainstem (blue arrows) causes contusion from contact with the meninges (XX) or the foramen magnum (X), usually with lethal consequences. Elevated plasma osmolality (right panel) draws water from the CSF. Under acute hyperosmolality, the brain sinks within the cranial cavity (downward magenta arrows). This can cause herniation through the foramen magnum (X) or longer-term demyelination of pontine white matter (XXX) or other structures (i.e., tegmentum, thalamus, putamen, caudate nucleus). Reduced brain flotation can stretch and damage cortical vessels (O). Overcorrection of plasma osmolality can occur by rehydrating from a hyperosmolar state using water instead of isotonic saline infusion. Conversely, a too rapid mannitol infusion in response to swelling can draw excessive water from the brain [13, 15]. Figure by J. A. Hellas. CSF, cerebrospinal fluid, SIADH, syndrome of inappropriate antidiuretic hormone.

Generally, membrane transport is categorized as (1) passive diffusion, (2) facilitated transport (by channels or carriers termed transporters), and (3) active transport, requiring energy. Osmotic swelling relies on transmembrane osmotic gradients [8] to draw water slowly and passively across the membrane, but is greatly facilitated if the membrane contains embedded water channels (aquaporins). Osmotic swelling occurs within tens of seconds upon exposure to hypoosmotic artificial cerebrospinal fluid (aCSF) [9]. Andrew et al. [2] proposed that most neurons lack functional aquaporins, supported by their minimal expression in neurons [10], and by RNA sequencing data showing that aquaporins are expressed in astrocytes [11] but not neurons [12]. Importantly then, acute hypoosmotic swelling of the brain is considered primarily astrocytic [5]. The gray matter behaves like an osmometer following sudden, persistent changes in plasma osmolality (Fig. 1) with the neurons as minor participants. For example, in an overhydrated distance runner before and during a race, water moves from gut, to blood, to cerebrospinal fluid (CSF), and finally into astrocytes. This hypoosmotic state causes the brain to swell, particularly in individuals with a compromised ability to resorb water by the kidneys. This syndrome of inappropriate antidiuretic hormone (SIADH) secretion results in dilute plasma and CSF, but also results in normal urine osmolality, causing disorientation, seizures, and even death (Fig. 1) [13, 14]. Conversely, a severely dehydrated runner requires isotonic saline infusion, as pure water infusion may induce too rapid an uptake with a similar outcome. This illustrates that osmotically driven water quickly moves between tissue compartments. Although neuronal volume is not significantly affected by acute deviations from normal plasma osmolality between − 60 and + 60 mOsm (the normal value in humans and rats is ~ 288 mOsm), brain excitability is affected by such shifts (“An Acute Drop in Osmolality Increases Excitability of the Live Brain Slice” section). Note that osmolality (mOsm/kg) is shortened here to mOsm.

In addition, the central nervous system (CNS) can be divided into three compartments composed mainly of incompressible liquids: blood (arterial and venous), brain parenchyma (intracellular and extracellular), and CSF. The Monro–Kellie doctrine posits that the sum of these volumes is constant; thus, an increase in one compartment decreases one or both of the other two. As such, the swelling of brain cells can dangerously reduce vascular volume. Furthermore, severe swelling can downwardly displace the brain, causing gray matter herniation and vascular damage (Fig. 1) [6, 13, 16, 17]; thus, a sudden reduction in plasma osmolality is dangerous in several ways. As an analogy, CNS neurons and the glial network behave as a hand holding a sponge: with acute hypoosmolality, astrocytes quickly swell like a sponge submerged in water, whereas the neurons (the hand) maintain their volume. Nevertheless, glial swelling alone can be fatal.

### An Acute Drop in Osmolality Increases Excitability of the Live Brain Slice

**Fig. 2:**
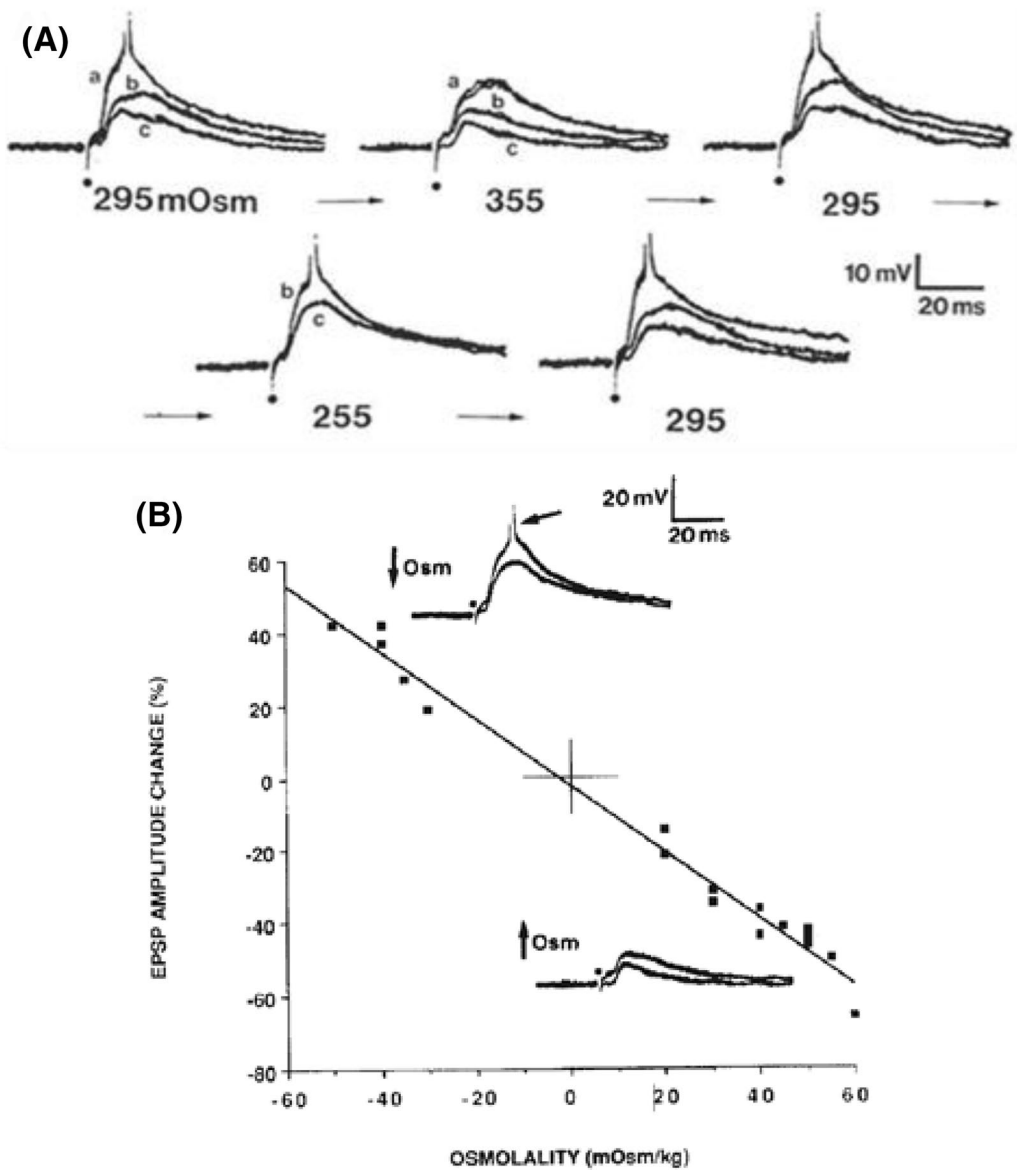
In pyramidal neurons recorded intracellularly from layers II-III in neocortical slices, the amplitude of evoked excitatory postsynaptic potentials (EPSP) varies inversely with aCSF osmolality. **a** EPSPs evoked by white matter stimulation (dot) [22]. The action potential stimulus threshold (T) in control saline (295 mOsm) evokes waveform “a.” Subthreshold stimuli evoke waveforms “b” and “c.” A 10 min change to hyperosmotic saline (355 mOsm) reduces EPSP amplitude at each stimulus strength. Control saline then completely reverses the effect over 10 min. On 10 min exposure to hypoosmotic saline (255 mOsm), EPSP amplitudes increase; the new threshold response is at 0.66 T. Again, control saline reverses the effect [22]. **b** The two insets show two evoked EPSPs from the same recorded neuron [23]. Hypoosmolality (− 50 mOsm) increases the amplitude of EPSPs, one driving the pyramidal neuron to fire an action potential (arrow, peak clipped). Hyperosmolality (+ 50 mOsm) reduces synaptic responses evoked at the same stimulus strength. Note that the regression line passes near the origin as expected for an inverse linear relationship between synaptic excitability and the osmolality. From R. D. Andrew [13]. aCSF, artificial cerebrospinal fluid, EPSP, excitatory postsynaptic potential, kg, kilogram, mOsm, milliosmole, ms, millisecond, mV, millivolt, Osm, osmole.

**Fig. 3:**
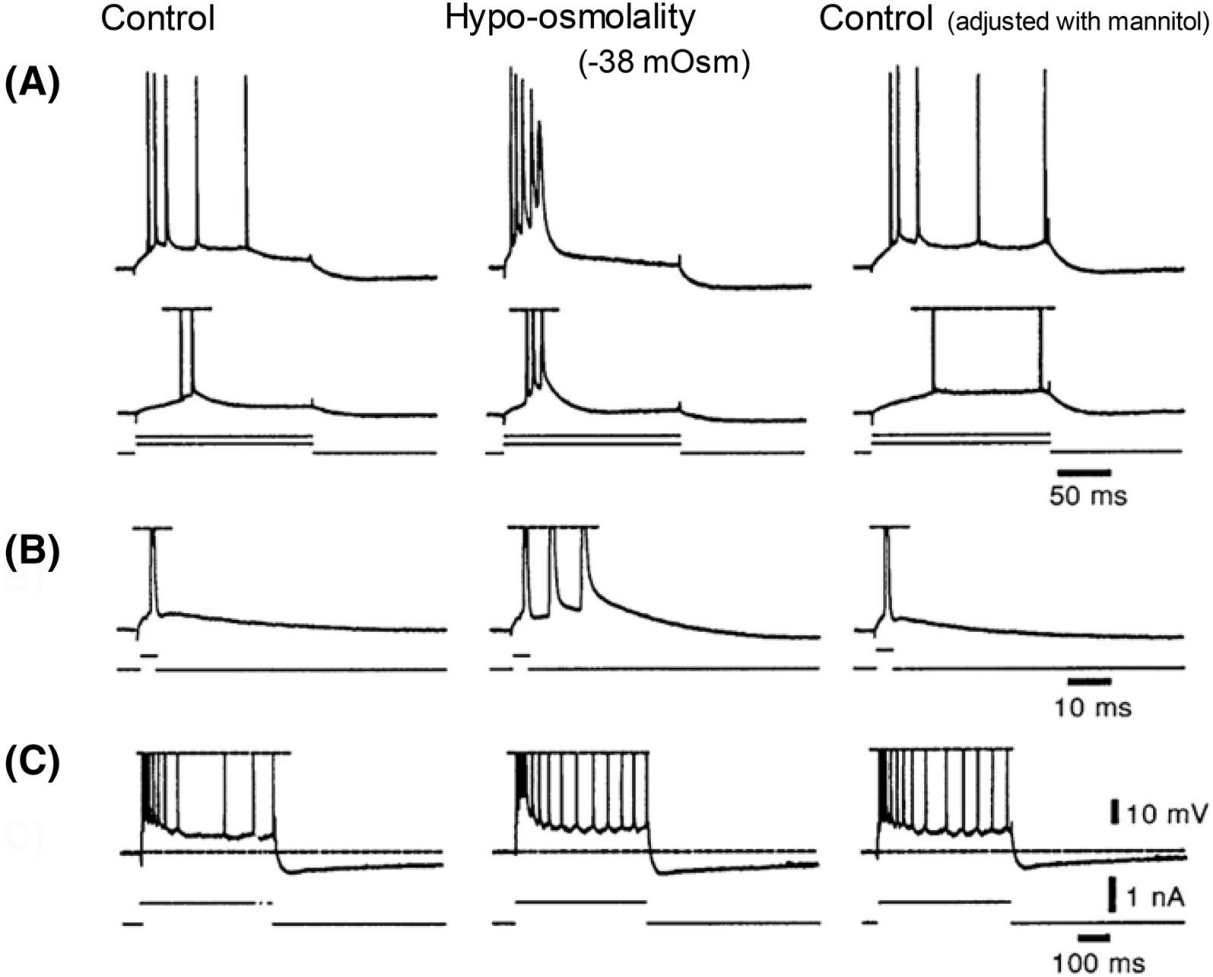
Enhancement of burst firing by low osmolality (− 38 mOsm) on a firing CA1 pyramidal neuron (resting potential, − 68 mV) in a slice of rat hippocampus. In normosmotic aCSF (control panels on the left), the cell displays no burst characteristics when stimulated with long (**a**) or brief (**b**) positive current pulses. In hypoosmotic aCSF (panels in middle) the cell displays burst firing in response to long (**a**) and brief (**b**) current pulses. This effect recovers after correcting to normosmolarity with mannitol (panels on right). The medium and slow AHPs are not affected by the hypoosmotic treatment (**c**). Hyperosmotic aCSF reduces burst firing (not shown). Modified from Azouz et al. [29]. aCSF, artificial cerebrospinal fluid, AHP, afterhyperpolarization, mOsm, milliosmole, ms, millisecond, mV, millivolt, nA, nanoampere.

As first described by Rowntree in 1923 [18], an acute reduction of plasma osmolality elicits disorientation and seizure activity in patients or experimental animals, independent of any physical brain damage noted above [13, 15]. Clinical examples include exercise-associated overhydration, hastened rehydration therapy, dialysis disequilibrium syndrome, or compulsive polydipsia. Each can become a clinical emergency if coupled with SIADH, the syndrome of inappropriate (i.e., low) antidiuretic hormone secretion [13, 14, 19–21]. Studies using brain slices have determined that several mechanisms promote elevated excitability and synchronicity when plasma becomes acutely hypoosmotic: (1) Increased synaptic strength, gauged by increased evoked excitatory postsynaptic potential (EPSP) amplitude and duration (Fig. 2) [22, 23], resulting from a concentrating effect on released transmitter as astrocytes swell and reduce the ECS volume. (2) Increased spontaneous EPSP frequency [22–24] synaptic responses, which are partly N-methyl-D-aspartate (NMDA) receptor-mediated [25]. (3) Increased field effects from reduced ECS volume, causing elevated extracellular resistance (R_e_), meaning that a fixed current (I) generated by a synchronized burst discharge will produce increased extracellular negative voltage (V_e_), from Ohm’s law (V_e_ = IR_e_). This adds to transmembrane potential, the depolarizing “blip” recruiting nearby silent neurons [26–28]. (4) Reduced ECS volume, which concentrates K^+^ and glutamate and further heightens excitation. (5) Altered intrinsic electrical properties of pyramidal neurons. Electrophysiological characteristics are subtly altered by acute osmotic change between − 80 and + 60 mOsm [22–24]. The amplitude of active spike afterdepolarization and burst excitability are increased by hypoosmotic treatment (the latter shown in Fig. 3). These osmotic effects are mediated by a persistent Na^+^ current and increased membrane time constant [29]. Both are enhanced by hypoosmolality, adding to burst excitability [29]. Hypoosmotic change also increases the membrane time constant, slowing the spike afterdepolarization, and thereby promoting additional spiking (Fig. 3) (Both properties are counteracted by hyperosmolality, a crucial requirement for convincingly demonstrating osmoresponsiveness, adhered to by each study noted above). The excitatory actions result from osmolality dilutions by as little as 13%. Working together, they elevate intrinsic neuronal excitability (slightly) and network excitability (considerably), thereby promoting increased likelihood of hippocampal and neocortical interictal bursting and seizure initiation over many minutes. However, over hours and days, as neuronal swelling begins, excitability dramatically decreases (“Neuronal Recovery and Water Loss” section).

Does acutely reduced osmolality induce SD in vivo? The answer seems to be not at clinically relevant pathophysiological levels. Highly unphysiological hypoosmotic media promoted SD in one study [30] but not in three others [2, 7, 31]. One in vivo study reported SD generation under very low osmotic conditions [32]. We know of no clinical reports in which a patient with acutely lowered plasma osmolality showed SD symptoms. Nevertheless, osmotic gray matter swelling might promote regional SD propensity by increasing tissue excitability via the mechanisms noted above.

### Long-Term Osmotic Brain Cell Volume Regulation

Subacute CSF osmolarity changes over hours or days are tolerated in vivo [9] via regulatory volume increases (RVI) or regulatory volume decreases (RVD). RVI counteracts initial passive hyperosmotic cell shrinkage, increasing cell volume to normal [3]. RVD involves the reverse pathway in response to hypoosmolality, acting to reduce cell volume [3, 33] through active ion and osmolyte efflux followed by water [33].

There is evidence supporting long-term RVD/RVI (hours and days) in experimental animals under osmotic stress [3, 34]. Brain cells can actively regulate volume in response to hypoosmotic stress over many hours by losing so-called organic solutes (also termed idiogenic osmoles), which are small molecules such as the amino acids glutamate and taurine [35]. In contrast, under hyperosmotic stress, organic solutes are synthesized to retain water and maintain volume. This volume regulation has been demonstrated by Arieff et al. [36] in animal models with hyperglycemia, in which brain tissue initially lost ~ 10% of its water (as measured by weight). However, after several hours, cerebral organic solute concentration was elevated to minimize water loss [36, 37], indicating adaptation through solute accumulation. It is unclear whether long-term RVI and RVD occur in glia, neurons, or both.

### Short-Term Brain Cell Volume Regulation

There is little evidence beyond isolated cell studies that brain cell RVI/RVD occurs over minutes following sudden osmotic challenge [2, 3]. This is important because short-term hypoosmotic conditions combined with SIADH can catastrophically swell the brain, which can be lethal due to the unyielding volume of the skull, spinal column, and meninges (Fig. 1) [3, 38, 39]. Worse, hypoosmolality promotes seizure activity (“An Acute Drop in Osmolality Increases Excitability of the Live Brain Slice” section), which in itself will promote brain swelling [3, 6, 40].

**Fig. 4:**
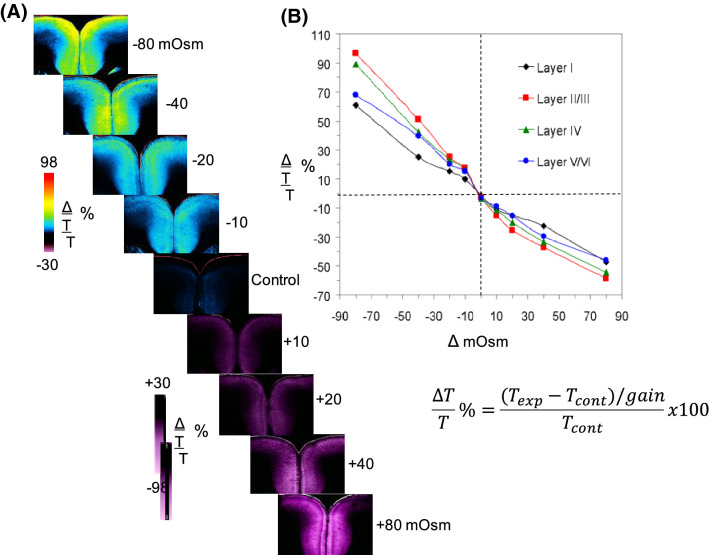
Direct relationship between altered light transmittance (∆LT) and osmolality. **a** ∆LT can be imaged through a brain slice to indirectly measure cell swelling or shrinkage. Inverse relationship between light transmittance and aCSF osmolality is imaged in a rat neocortical slice. Gray matter LT increases in hypoosmotic aCSF (− 10 to − 80 mOsm) and completely reverses within 8 min of return to control aCSF (not shown). Conversely, LT decreases as osmolality is increased using mannitol (+ 10 to + 80 mOsm). The signal is primarily astrocytic, given that neuronal volume resists osmotic shifts within this pathophysiological range. Unpublished figure. **b** Plot of the mean peak change in LT versus shift in osmolality from a control level of 287–289 mOsm [2]. Slices (*n* = 9) display an inverse relationship between change in osmolality and ∆LT in all neocortical layers. From Andrew et al. [2]. aCSF, artificial cerebrospinal fluid,  LT, light transmittance, ∆LT, change in light transmittance, ∆T, change in transmittance, mOsm, milliosmole.

There is an inverse linear relationship between light transmittance (LT) by gray matter of a live slice and the degree of osmotic shift (Fig. 4) because astrocytes behave as excellent osmometers [2, 41]. The optics are not detailed here. In short, swollen cell membranes adapt a more planar configuration which scatters less light, increasing LT. However, dendritic beads, although swollen, are of an optimum diameter to scatter light, thereby decreasing LT [42]. Confirming how astrocytes and neurons change their volume is facilitated by the real-time imaging afforded by two-photon laser scanning microscopy (2-PLSM).

**Fig. 5:**
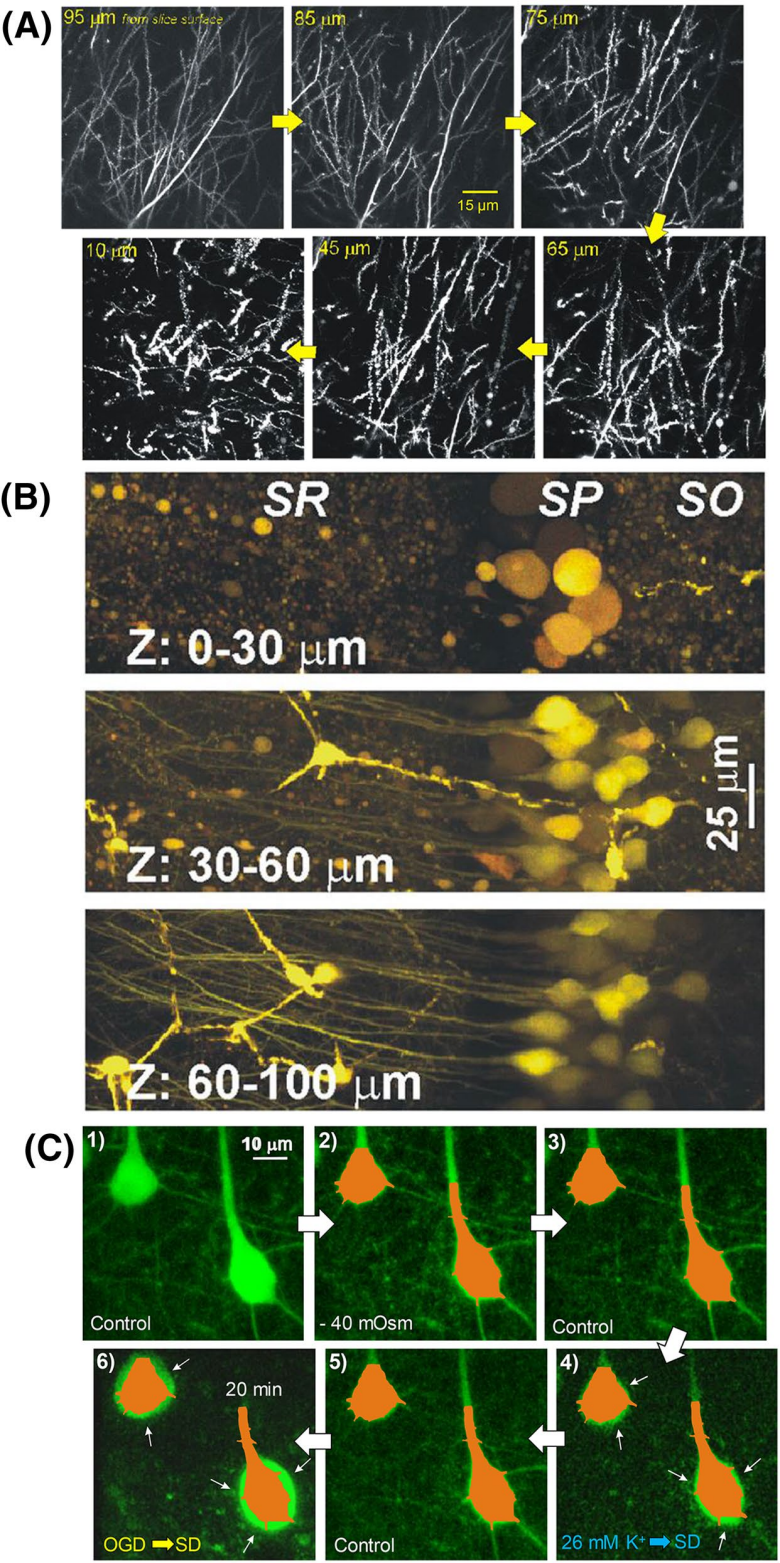
Pyramidal neurons are increasingly damaged and swollen closer to the cut surface of a live brain slice as imaged with 2-PLSM. Optical sections through the CA1 dendritic region. The mouse expresses eGFP in random pyramidal neurons. **a** Sections are captured at depths progressively closer to the slice’s cut surface. Closer to the cut surface (10 µm), there is increased dendritic swelling/beading as more branches of each CA1 arbor are cut. Images adapted from Davies et al. [47]. **b** Two-photon fluorescence images of neurons in the hippocampal slice scanned at successively deeper levels. Grossly beaded and swollen CA1 pyramidal neurons are imaged near the sliced surface. Neurons take on a normotypic structure with depth. Images from Dzhala et al. [48]. **c** Pyramidal cell bodies resist volume increases induced by osmotic stress but swell following SD. Masks representing the control volumes of two cell bodies somata in (C1) are placed over the same somata following subsequent treatments. Exposure to hypoosmotic aCSF (– 40 mOsm) reveals no increase in somata volume at 5–6 min (C2) or 15 min (not shown). Following return to control aCSF (C3), exposure to aCSF with 26 mM K^+^ for 2–3 min evokes cell body swelling (C4, arrows), which is reversible (C5). 20 min post OGD (C6), swelling is even more pronounced (arrows) and proximal dendrites fade. 2-PLSM, two-photon laser scanning microscopy, aCSF, artificial cerebrospinal fluid, eGFP, enhanced green fluorescent protein, mM, millimolar, mOsm, milliosmole, OGD, oxygen–glucose deprivation, SD, spreading depolarization, SR, stratum radiatum, SP, stratum pyramidale, SO, stratum oriens, µm, micrometer.

**Fig. 6:**
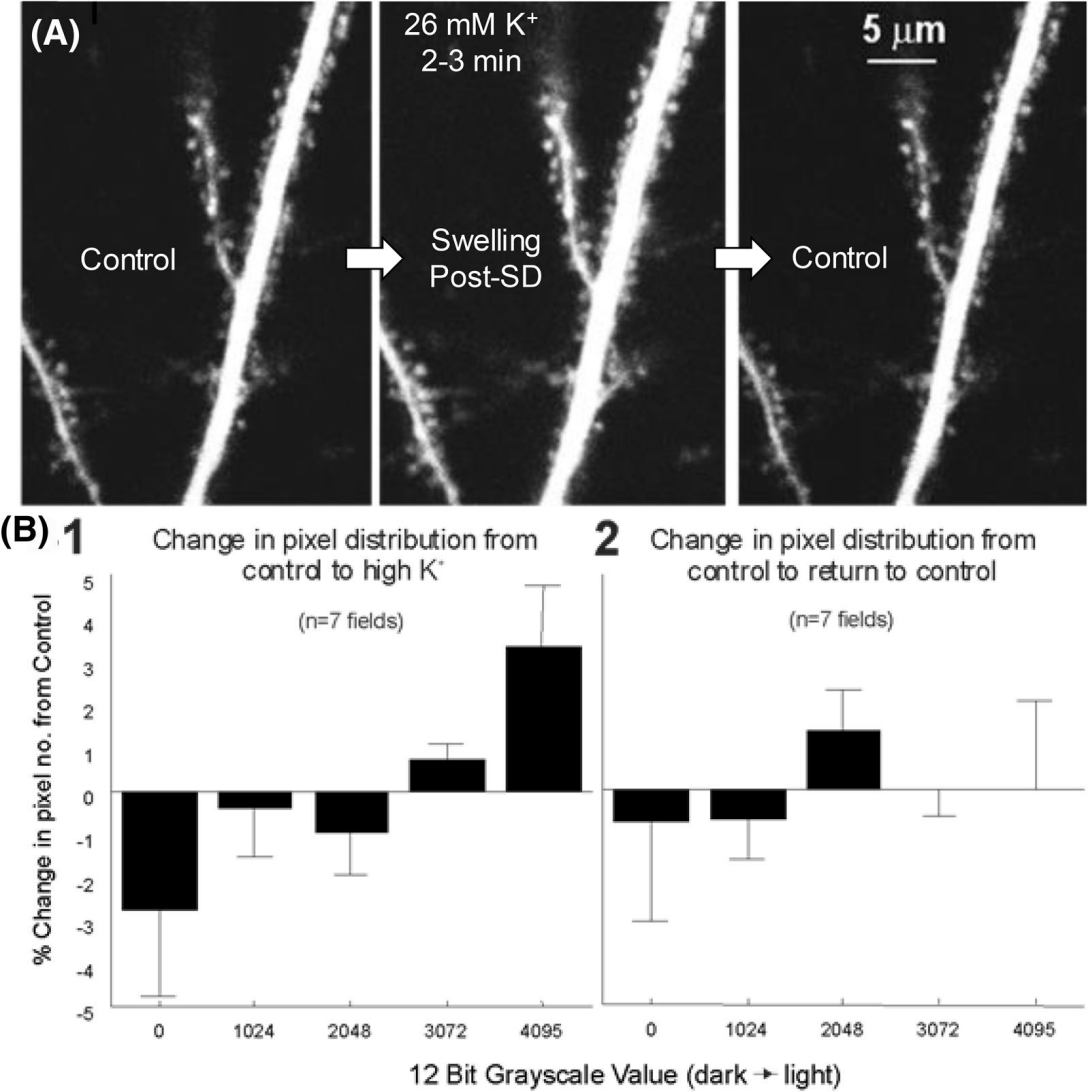
**a** Exposure to aCSF with elevated K^+^ evokes SD and reversible dendrite swelling with a return to control volume by 20 min as imaged with 2-PLSM. **b** Histogram of the mean change in pixel gray value relative to control for a pool of 4 somatic fields and 3 dendritic fields exposed to 26 mM K^+^ aCSF for 3–5 min. As signified by the recruitment of dark to light pixels, there is an expansion of neuronal volume in comparing control to high K^+^ aCSF (B1) but not in comparing control to return to control (B2). Thus, neurons and their dendrites swell reversibly during SD evoked by high K^+^. From Andrew et al. [2]. 2-PLSM, two-photon laser scanning microscopy, aCSF, artificial cerebrospinal fluid, mM, millimolar, SD, spreading depolarization, µm, micrometer.

A study by Murphy et al. [43] claimed evidence of neocortical neuron swelling under extreme hypoosmotic stress. Their confocal imaging detected reduced fluorescence intensity, presumably from cytoplasmic dilution of the fluorophore, as a measure of neuronal swelling. This contradicted several studies using 2-PLSM [2, 7], and their interpretation is compromised by several issues. First, they detected hypoosmotic swelling in neurons close to the slice surface (30–40 µm) where cells are damaged from slicing (Fig. 5a, b). For instance, Andrew et al. [2] noted that many neurons shallower than 50 µm displayed swollen cell bodies and beaded dendrites (Fig. 5a). Second, Murphy et al. [43] saw no cell body swelling further from the slice surface (65–90 µm), supporting other reports of no hypoosmotic swelling of neurons detected in deeper, healthier regions [2, 7] of up to 200 µm [44]. Third, at depths > 40 µm, confocal microscopy loses significant resolution compared with 2-PLSM, preventing accurate measurement of dendritic and axonal volumes at depth. Because these regions represent > 99% of pyramidal neuron membrane surface, it is prudent to measure more than just the somata. Andrew et al. [2] measured all three regions. As with neuronal soma (and unlike adjacent astrocytes), dendrites and axons > 50 µm below the slice surface steadfastly maintained their volume between − 40 and + 80 mOsm. Then, under OGD, Andrew et al. [2] imaged each neuronal region that displayed constant volume under acute osmotic stress (“Neuronal Swelling in Response to Ischemia” section). In every case, there was rapid and dramatic swelling evoked by SD (somata shown in Fig. 6). Thus, neurons that were consistently unresponsive to osmotic shifts were clearly healthy. In fact, SD is the standard cause of neuronal swelling detected in 2-PLSM studies, whether induced by high K^+^ [2, 45], OGD [2, 7], or in vivo by ischemia [46].

Finally, the suggestion by Murphy et al. [43] that Andrew et al. [2] failed to detect neuronal swelling because of higher temperature (35 °C) or the aCSF not reaching deeper neurons is without merit because adjacent astrocytes at depth responded with appropriate osmotic volume changes. Likewise, Hirrlinger et al. [49] observed astrocytic osmotic swelling at depths > 40 µm, and swelling continued for 37 minutes, increasing almost linearly. Evidently, increased depth does not hinder aCSF perfusion or the observation of swelling.

In addition, no neuronal RVD or RVI was observed. Neither was RVD nor RVI detected in adjacent, osmotically-compliant astrocytes [2, 7]. We conclude that neurons do not exhibit detectable volume responses, nor short-term volume regulation under acute, physiologically relevant, osmotic shifts.

### Osmosensory Neurons are Apparently Osmoresponsive

One type of mammalian neuron apparently displays osmoresponsiveness, although there is only minimal documentation that these neurons volume-respond in studies using nonisolated cells [50]. These neurons have long been theorized to intrinsically sense plasma osmolality changes by swelling and shrinkage during hypoosmotic and hyperosmotic conditions, respectively. For example, magnocellular neuroendocrine cells in the supraoptic and paraventricular hypothalamic nuclei increase firing with slightly elevated plasma osmolality [51], triggered by the shrinkage-induced opening of transient receptor potential vanilloid type-1 channels. This causes depolarization and discharge, promoting pituitary antidiuretic hormone (vasopressin) release, which signals renal water conservation. Other leaky BBB regions may also facilitate hyperosmolality detection by osmosensitive neurons, causing the perception of thirst [52, 53]. Similarly, astrocytic shrinkage for sensing hyperosmolality in these hypothalamic regions is important for thirst detection [54]. Notably, however, osmosensitive neurons have not been reported to express aquaporins.

### Questioning Classic Assumptions of Osmotic Theory

Aquaporin-4 (AQP4) is expressed abundantly in glia, particularly astrocytic endfoot processes abutting blood vessels, ependymal cells, and the subarachnoid space lining [55, 56] ("Brain Cell Swelling with Osmotic Shifts" section). AQP4 expression is upregulated in several pathologies, including ischemia and TBI [57], and is known to significantly mediate cerebral edema through glial swelling [8, 57, 58]. Solenov et al. [57] observed reduced astrocytic swelling in AQP4-deficient mouse models. Although some glial osmoresponsiveness has been observed with AQP4 inhibition [59], this may be due to other membrane transporters passively moving water, providing residual permeability [8]. Although AQP4 expression is a major contributor to SD-associated glial swelling, it is not an absolute requirement, nor are aquaporins needed for neuronal osmosensitivity (“Osmosensory Neurons are Apparently Osmo-responsive” section).

#### Barrier Membranes

Lipid membranes lacking aquaporins are relatively water-impermeant, although some does permeate. Permeability is further reduced if the outer leaflets have less viscous lipid components (i.e., high cholesterol content and tightly-packed fatty acid tails) [60], conferring high microviscosity. Whether neuronal membranes possess properties of these so-called barrier membranes [60] has not been studied.

#### Water “Pumps”

Water can be actively transported across membranes both under isoosmotic conditions [61], and even against osmotic gradients [62, 63] (“Transport (Carrier) Proteins and Neuronal Swelling” section).

**Table:** Box 1 The waterlogged human brain

*Water intoxication (WI)*. WI is an older term referring to the disorientation and loss of consciousness (often with generalized seizure) that develops in response to excessive water drinking. WI develops when plasma osmolality acutely drops by 20 mOsm or more from a normal level of ~ 288 mOsm [13, 18, 15]. In each of the situations described below, undiagnosed SIADH in the individual is often a major contributor to the inadequate diuresis that results in dilute plasma and CSF. While hyponatremia invariably results, excess water is the more immediate concern because of brain swelling and associated hyperexcitability (“An Acute Drop in Osmolality Increases Excitability of the Live Brain Slice” section). The following are three causes of WI:
*Forced water drinking.* Each year, clinical case studies are published of individuals who imbibe excess water. The perception by the lay public is that such behavior is innocuous. The motivation includes carrying out a stunt or entering a contest for some reward [13]. A more common victim is the misbehaving child who is forced to drink excessive water as a punishment, often with lethal results [64]
*Compulsive water drinking (polydipsia).* Numerous studies have described compulsive water drinking by schizophrenic individuals. The institutionalized patient behaves normally in the morning, but with free access to a water fountain, becomes disoriented and may suffer a generalized seizure by the afternoon [13]
*Overhydration in endurance sports*. While it may seem conspiratory, hospitalizations of marathoners and triathletes were rare prior to the establishment of the sports drink industry in the 1970s [14]. Their promotion of imbibing fluids both before and during endurance races (supposedly to replace sodium lost from sweating) has resulted in death for numerous athletes. These victims were not hyponatremic. Rather, their symptoms were those of overhydration specifically: headache (uncommon with severe dehydration), bloating and swollen extremities, nausea and vomiting (from increased intracerebral pressure), generalized seizure [14]. Note that seizure is not the result of increased intracerebral pressure or brain “irritation” caused by expansion, but rather by elevated neuronal excitability as described in “An Acute Drop in Osmolality Increases Excitability of the Live Brain Slice” section [13]. For the past 15 years, the simple advice to endurance athletes has been the following: do not water load, drink when you are thirsty

## Brain Cell Swelling and Ischemia

### Neuronal Swelling in Response to Ischemia

Neuronal swelling during ischemia is classically considered osmotically driven. The textbook explanation of cytotoxic edema describes a premorbid process, whereby extracellular Na^+^ enters neurons through voltage-sensitive Na^+^ channels activated by ischemia. Na^+^ accumulates intracellularly, exacerbated by the Na^+^/K^+^ pump’s reduced ability to extrude Na^+^ [4]. Negatively charged intracellular proteins hold in K^+^ via Donnan forces. Na^+^ and K^+^ buildup, then somehow electrostatically draw in Cl^−^. Intracellular accumulation of all three ions then purportedly draws in water by osmosis through some undefined conduit and elicits neuronal swelling [65].

But is this scenario correct? Let us begin with the initial blood flow loss that lowers tissue ATP levels. This slows the Na^+^/K^+^ pump, causing some intracellular Na^+^ accumulation. Neurons may then respond with hyperpolarization for several seconds generated by an ATP-sensitive K^+^ current and/or by Na^+^/K^+^ pump upregulation [66]. Next follows a depolarizing phase (accompanied by spike inactivation presumably from inhibition of voltage-sensitive Na^+^ channel opening, although those channels contribute some inward Na^+^ current). As the Na^+^/K^+^ pump completely fails, a precipitous opening of a massive and nonspecific Na^+^/K^+^ conductance [67] initiates SD which resists glutamate receptor antagonists and tetrodotoxin (TTX) blockade. Prior to SD, the K^+^ transmembrane gradient ([K^+^]_i_ = 155 mM; [K^+^]_o_ = 4 mM) is opposite, although similar to the Na^+^ gradient ([Na^+^]_i_ = 12 mM, [Na^+^]_o_ = 155 mM). During SD, the membrane is freely permeable to Na^+^ and K^+^ which move down their concentration gradients. There is minimal net cation influx because Na^+^ influx is counteracted by K^+^ efflux. Dogma holds that Na^+^ influx exceeds K^+^ efflux because negatively charged intracellular proteins hold in K^+^, but entering Na^+^_i_ can play the same role, allowing K^+^ efflux. As well, voltage-sensitive K^+^ channels should open during SD, further increasing K^+^ conduction outward. So, there is no obvious cationic gradient drawing in Cl^−^. Although chloride could simply move inward down its concentration gradient ([Cl^−^]_i_ =  ~ 7 mM; [Cl^−^]_o_ = 140 mM) [68], Cl^−^ influx and water uptake during ischemia involves specific transporters (“Transport (Carrier) Proteins and Neuronal Swelling” section) rather than ligand-gated channels activated by γ-aminobutyric acid (GABA) or glycine. From experiments substituting extracellular Cl^−^ with other anions, Cl^−^ influx is not required for SD generation, but it is required for neuronal swelling and dendritic beading post-SD [31, 69]. Yet ischemic swelling and beading of neurons are considered independent of osmotic gradients [8, 62, 63] (“Osmosensory Neurons are Apparently Osmo-responsive” section).

**Fig. 7:**
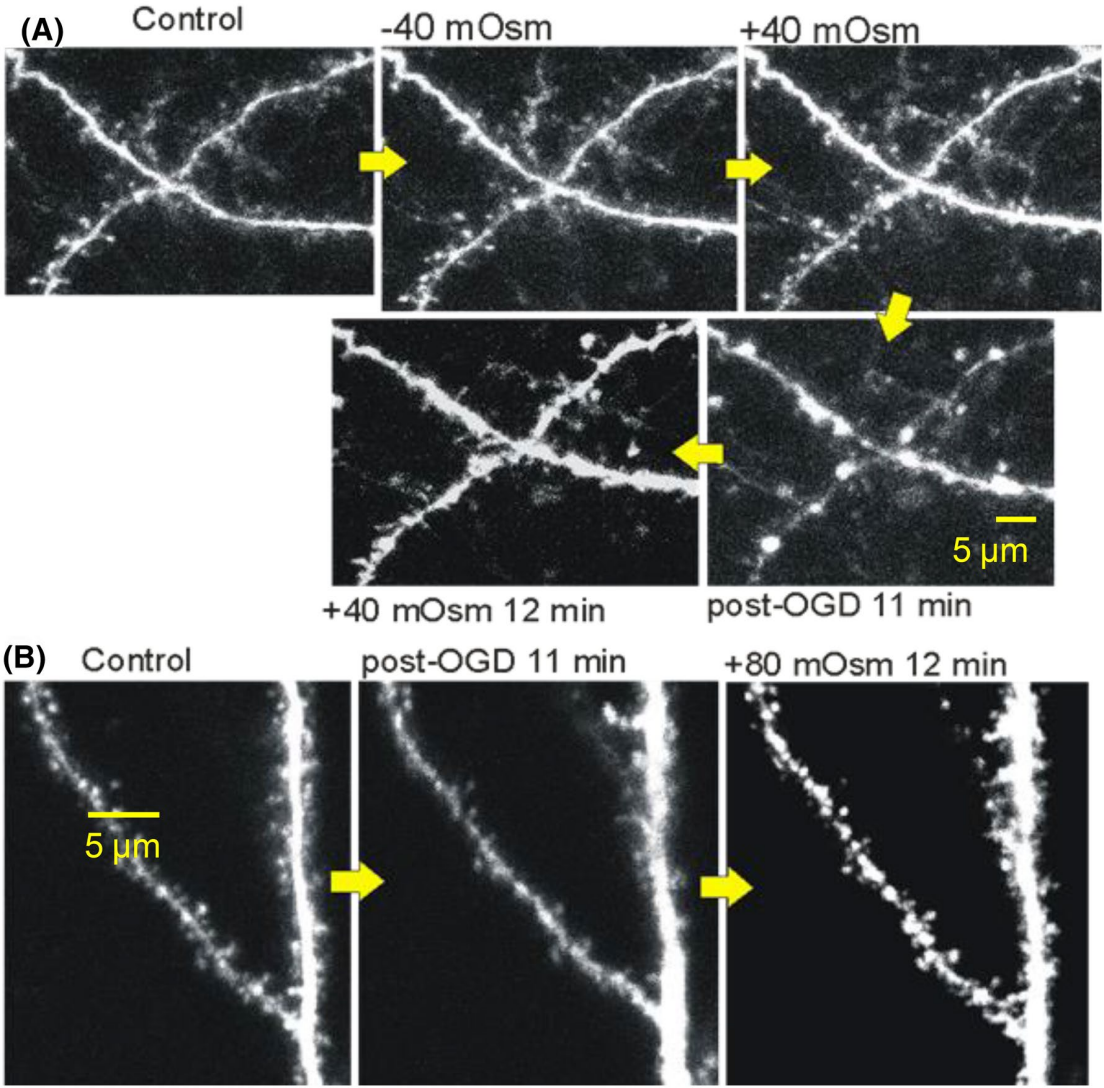
2-PLSM optical sections through the same CA1 dendritic region. **a** Neither increased nor decreased aCSF osmolality by 40 mOsm for 10 min alters dendrite volume. OGD induces ongoing dendritic swelling/beading which continues for 11 min post-OGD, resulting in swollen dendrites with beads up to 5 µm in diameter. Return to hyperosmotic aCSF (last panel) does not reduce swelling. **b** OGD induces dendritic swelling/beading which is not counteracted by 12 min of hyperosmotic aCSF exposure. Images modified from Andrew et al. [2]. 2-PLSM, two-photon laser scanning microscopy, aCSF, artificial cerebrospinal fluid, mOsm, milliosmole, OGD, oxygen–glucose deprivation, µm, micrometer.

This begs the question: What drives neuronal swelling in the wake of SD? Inhibiting the cotransporters NKCC1 and KCC2 (chloride transporters), anion exchange protein (AE3), and monocarboxylate transporter (MCT2) reduces dendritic beading in vivo, indicating that pronounced ion displacement during SD leads to transmembrane solute imbalances, dramatically reducing neuronal water influx in the seconds and minutes post-SD [31] (“Transport (Carrier) Proteins and Neuronal Swelling” section). Over the longer-term, the NKCC1 blocker bumetanide reduces cerebral edema in vivo post-TBI by decreasing cotransported water influx from stressed neurons [58]. Is this swelling osmotically driven? Unpublished experiments by Andrew and Kirov associated with a previous publication by Andrew et al. [2] show that post-SD slice swelling cannot be countered by simple exposure to hyperosmolar aCSF lasting 10–15 minutes (Fig. 7b). Post-SD, neuronal expansion is ongoing and continuous over a minute or more, to the point where cell bodies become swollen spheroids. Additionally, dendritic bead diameter can approach 5 µm (Fig. 7a).

**Fig. 8:**
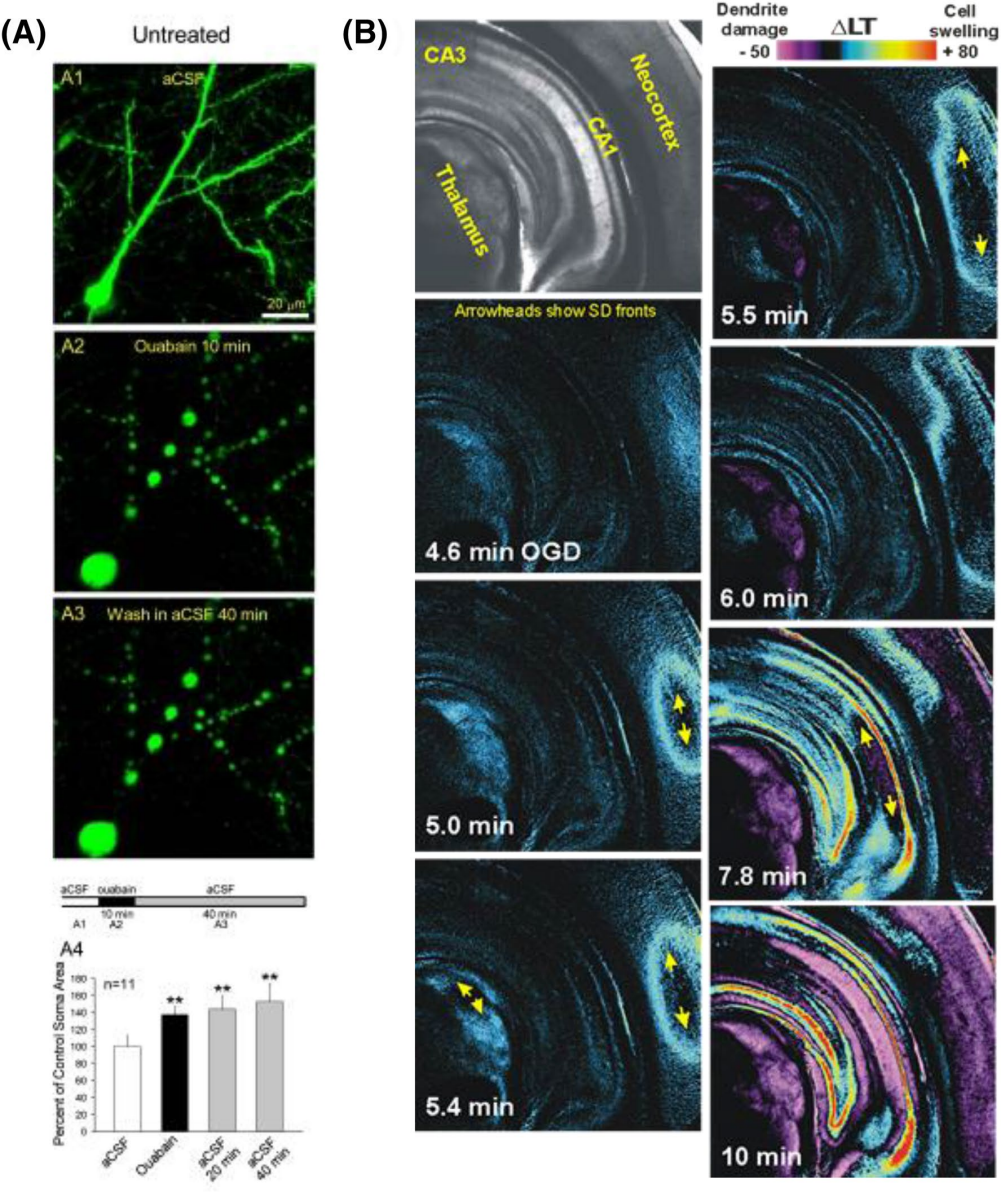
**a** The cell body of an eGFP-expressing pyramidal neuron prior to SD (A1) swells following SD evoked by 100 µM ouabain for 10 min (A2). Ouabain is a Na^+^/K^+^ pump inhibitor that induces SD. Swelling does not recover, nor is there dye leakage during next 40 min in aCSF (A3). The horizontal bar (bottom panel) shows the time course of the experiment and indicates when images A1–A3 were acquired. A4) Summary from 11 neurons in 9 slices from 7 animals showing irreversible soma swelling induced by 10 min of superfusion with 100  µM ouabain. Values are shown as percent of control. Shading of each histogram bar corresponds to the same shading in the time line bar above. Asterisks indicate significant difference from control. From Douglas et al. [72]. **b** SD propagation through higher brain regions, indicated by changes in light transmittance (LT) imaging. A coronal brain slice from adult rat is exposed to OGD for 10 min at 34 °C. SD is generated in the neocortex (5 min) and thalamus (5.4 min), as well as in the CA1 region of hippocampus (7.8 min). Neuronal and astrocytic swelling arises at each moving SD front. Then over several minutes, dendritic beading develops which increasingly scatters light in the wake of SD (purple pseudocoloring, 10 min). Modified from Brisson et al. [73]. aCSF, artificial cerebrospinal fluid, LT, light transmittance, µM, micromolar, ∆LT, change in light transmittance, eGFP, enhanced green fluorescent protein, SD, spreading depolarization. **P 0.005.

Innumerable injured neurons near the cut slice surface do not lyse over many minutes (Fig. 8a) or even hours (Figs. 5a, b), indicating that a cytosolic water-osmolyte equilibrium must be reached. Neuronal volume can increase severalfold post-SD, so likely more than Na^+^_i_, K^+^_i_, and Cl^−^_i_ are holding in water. Degradation of nondiffusible proteins (or any large molecules) into their constituent parts may increase cytosolic osmolality according to Gibbs–Donnan equilibrium, which would hold water intracellularly [70]. However, it is unclear what percentage of such water would be “restricted.” Restricted water is constrained to the hydration layers of macromolecules (reviewed in [68]). The layers of restricted water are only a few molecules wide, but the density of macromolecules in the cytoplasm is high. Thus, while the amount of restricted water is ~ 50% of all cytoplasmic water, it cannot contribute to the intracellular osmolality [68]. So, it is unclear to what degree macromolecular breakdown would draw in water. Alternately, the role of membrane stretching and/or increased hydrostatic pressure on swelling dynamics is difficult to model, but it has been proposed that increased intracellular pressure from the stretched plasma membrane could reduce water influx [71], which might stabilize the grossly swollen state of cell bodies and dendrites.

An older definition of cytotoxic edema described intact capillaries as the source of water and electrolyte (termed ionic edema). However, it is apparent that a major source is CSF inflow to the quickly-expanding perivascular space (PVS) from SD-evoked propagating vasoconstriction [74]. This propagating ‘spreading ischemia’ immediately follows in the wake of SD, as do astrocytic and neuronal swelling. Astrocyte swelling is understood to result from K^+^ taken up from depolarized neurons, followed by osmotic Cl^−^ uptake, and selective water uptake via AQP4. Note again, AQP4’s recently-appreciated role in SD [8, 62].

Another contributing factor to neuronal swelling may be metabolic water, a product of oxidative metabolism (specifically, oxidative phosphorylation). Total mammalian neuronal metabolic water is estimated from different models to be 7–56%, although it was accurately measured by Li et al. [75] in bacteria to be 30–40%. We suggest that during ischemia, residual oxidative metabolism may generate intracellular water, enhancing swelling.

Neuronal swelling can also be artificially-elicited with neurotoxins that open Na^+^ channels, thereby pathologically increasing [Na^+^]_i_. For instance, veratridine opens Na^+^ channels, eliciting Na^+^ influx and SD [76] which swells neurons in brain slices [65]. Glutamate receptor agonists like NMDA [65] or domoic acid [77] also swell neurons in slices without evoking SD. Blockers of the voltage-gated Cl^−^ channel, SLC26A11, reduced neuronal swelling from veratridine and NMDA in slices, but had little effect on SD onset time or neuron swelling [31, 65, 78]. These studies have more relevance to neurotoxicity than to ischemia or SD.

### SD is the Common Cause of Acute Neuronal Swelling

Except for neurotoxic poisoning (previous paragraph), ischemia-induced SD caused by stroke, TBI, subarachnoid hemorrhage, or sudden cardiac arrest promotes acute neurological damage. Specifically, neuronal swelling and beading is initiated within seconds of SD invading imaged gray matter [79]. Recently, it has become clear that SD itself expands the PVS, exacerbating brain swelling. In mouse stroke models, a single SD event evokes propagating vasoconstriction, expanding the PVS [74]. Water entering the PVS from CSF accesses astrocytes through endfoot AQP4, contributing to early poststroke cytotoxic edema. How neurons swell is more mysterious (“Neuronal Swelling in Response to Ischemia”, “Transport (Carrier) Proteins and Neuronal Swelling” sections). Importantly, cytotoxic edema is a direct result of SD, yet SD is not even mentioned in many recent reviews of neuronal death [80–83].

Aquaporins are expressed in astrocytes, accounting for their osmoresponsiveness (“Short-Term Brain Cell Volume Regulation” section). Table 1 provides an overview of aquaporin isoform expression by brain cell type [10, 56, 57, 84–90]. Astrocytes swell with hypoosmolality and shrink with hyperosmolality within seconds, while most neurons do not [2, 7, 16]. Neurons resist osmotic swelling (Figs. 5c, 7a) or shrinkage (Fig. 7a). So, astrocytes are the primary cellular contributor to cerebral edema in an acute dilutional situation (Fig. 1) commonly induced by a behavioral or iatrogenic event (Box 1).

**Table 1:** Aquaporin expression by brain cell type

Brain cell type	Aquaporin type
Astrocyte	AQP3, AQP4, AQP5, AQP8, AQP9 [57, 84–86]
Oligodendrocyte	AQP8 [85, 87]
Ependymal cell	AQP1, AQP4, AQP8, AQP9 [56, 84, 87–89]
Microglia	AQP4 [87, 90]
Neuron	AQP1^a^, AQP9 [10]

*AQP,* Aquaporin

^a^It should be specified that these are only in dorsal root ganglion neurons [10]

In contrast, neurons start to swell within seconds following SD in situ induced by ischemia [2, 91, 92], or in slices induced by elevated [K^+^]_o_ (Figs. 4, 5B3), OGD [2] (Figs. 5b5, 6), hyperthermia, or hypothermia [93, 94]. In all cases, SD is necessary and sufficient to evoke neuronal swelling. The mechanism of this swelling is poorly-understood, although the common denominator is likely Na^+^/K^+^ pump dysfunction [95].

#### OGD-Induced SD

Bath-applied OGD quiets neuronal firing over 3–5 minutes prior to SD initiation even though a small, slow baseline depolarization may be generated. In “higher” brain neurons (those not in the hypothalamus or brainstem), SD onset is sudden with minimal discharge, and follows rapid depolarization toward 0 mV (Fig. 6b) with dramatic swelling of astrocytes *and* neurons over the ensuing minutes (Figs. 5b5, b6, 6a). Elevated LT representing the SD front propagates through higher gray matter (Fig. 8b, light blue). Dendritic swelling evolves to “beading” after ~ 6 minutes depending on dendrite diameter (Fig. 8b) [91]. This scatters light, thereby reducing LT (Fig. 8b, purple) and demarcating the dendritic dysmorphia arising from SD. The prerequisite of SD for neuronal swelling is nicely illustrated by hypothalamic [96] and brainstem neurons [73] which only weakly undergo OGD-evoked SD. These neurons show no detectable swelling, and fully recover electrophysiologically.

SD induced by Na^+^/K^+^ pump inhibitors such as ouabain or palytoxin results in typical neuronal swelling/beading within minutes. Likewise, SD from other treatments that stress the Na^+^/K^+^ pump (hyperthermia or hypothermia) also swell higher brain neurons (not detailed here).

#### [K^+^]_o_-Induced SD

Elevating [K^+^]_o_ focally or by bath elicits SD in slices of the higher brain, but not of brainstem or hypothalamus [79]. As with other ways of inducing SD, high [K^+^]_o_ likely inhibits the Na^+^/K^+^ pump, although the exact mechanism is still unclear [95]*.* Briefly increasing [K^+^]_o_ to 26–40 mM in slices induces pre-SD astrocytic swelling, primarily from neuronal discharge and astrocytic K^+^ uptake, with water osmotically following [45, 97]. It is not until bath-applied K^+^ depolarizes neurons (according to Nernst’s equation for K^+^) by at least 20 mV that channels open, generating SD [79]. Further, once SD is evoked by brief KCl application, neuronal cell bodies briefly and reversibly swell (Fig. 4, 5b3), as do their dendrites (Fig. 6). As noted by Rungta et al. [65], cortical neuron depolarization with 40 mM [K^+^] is insufficient to cause swelling until SD is generated, concurrent with depolarization near 0 mV and with substantial [K^+^]_o_ accumulation [45, 98].

### Neurons May Remain Swollen Post-ischemia, Exhibiting Reduced Excitability

The intact brain becomes hyperexcitable in response to acute osmotic swelling of the astrocytes (“An Acute Drop in Osmolality Increases Excitability of the Live Brain Slice” section). This can induce generalized seizure in vivo over tens of minutes, particularly in cases of undiagnosed SIADH [13–15]. This excitability arises at the cellular level and is not a result of brain compression. Over many hours following stroke or TBI, neurons may join astrocytes in swelling, but these neurons display lowered excitability [58, 99].

**Fig. 9:**
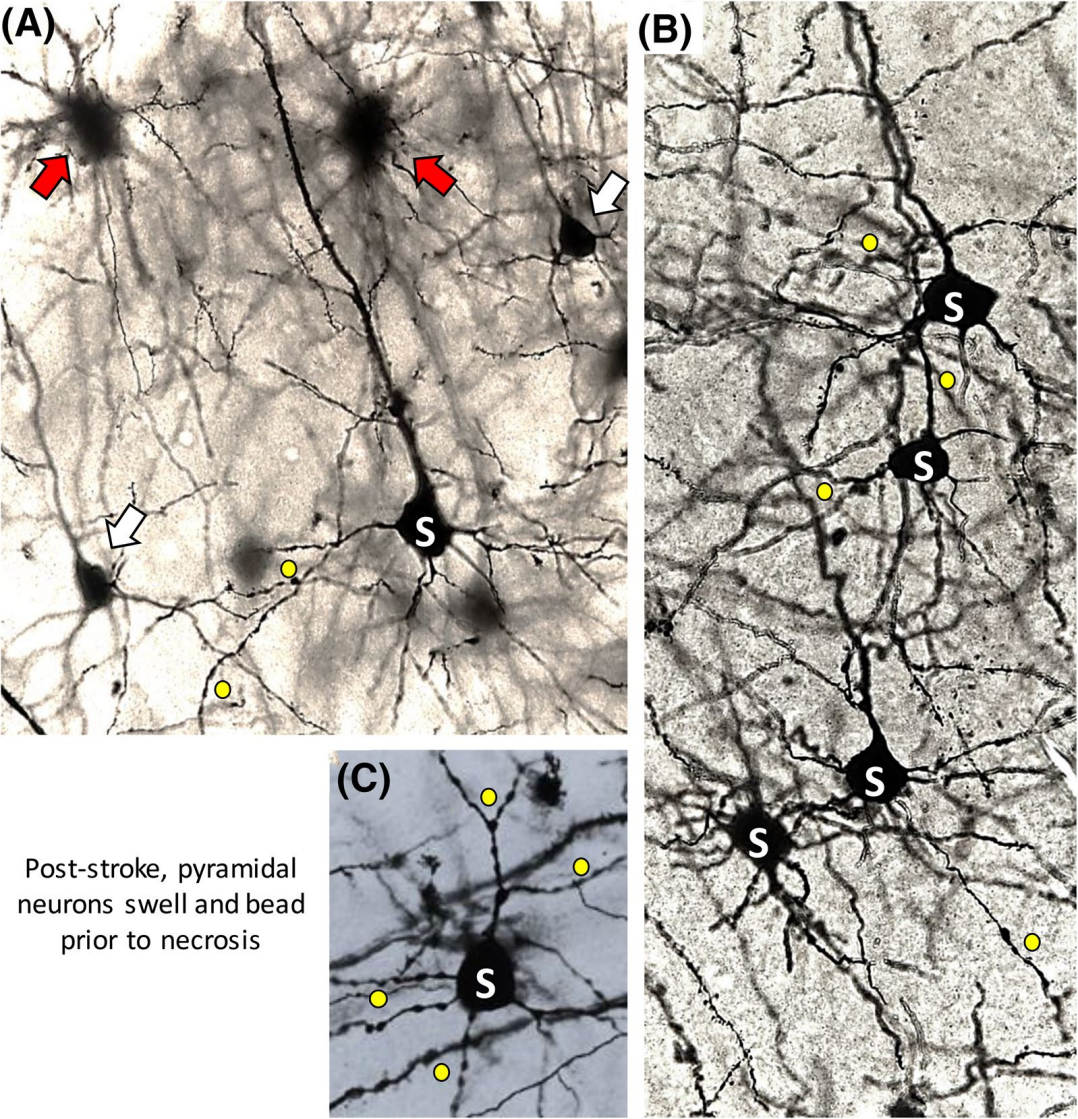
Different stages of ischemic injury to neurons poststroke. Neurons stained by the Golgi-Cox method are imaged near the territory of the ischemic core in the mouse neocortex 12 hours post-MCAo. Neurons may be structurally unaffected (white arrows) or exhibit ischemic stress with swollen cell bodies (S) and dendritic beading (yellow dots). They may be severely necrotic with retracted dendrites imparting a spidery appearance (red arrows). Unpublished images from Petrin et al. [99]. MCAo, middle cerebral artery occlusion.

Twelve hours after middle cerebral artery occlusion (MCAo) in mice, there are subsets of pyramidal neurons near the infarct that are necrotic (red arrows, Fig. 9a), whereas others appear normal (white arrows, Fig. 9a). Many other pyramidal cells display intermediate disruption (i.e., swollen cell bodies) (S, Fig. 9a–c) and beaded dendrites (yellow circles, Fig. 9a–c) presumably the result of at least one SD event. These fields display reduced excitation electrophysiologically (not shown) [99]. Similarly, adjacent to a TBI lesion, swollen neurons are observed in cortical regions exhibiting reduced excitability when patch-clamped [58]. So, ischemic edema swells neurons and astrocytes as well as reducing excitability over many hours or even days. This contrasts with acute osmotic swelling where neuronal and network excitability increases without neuronal volume changing.

### Hydration Shells, Channels, and Water Movement

Ions may move with or without their hydration shells while being conducted through ion channels. Most K^+^ channels completely strip each K^+^ ion of its water shell before passage. In the past, it was thought that Na^+^ ions were completely stripped of their shells by the voltage-sensitive Na^+^ channel, but it may only be partial. Regarding glutamate receptor-mediated Na^+^ influx and K^+^ efflux, both ions remain partly hydrated as they pass through the channel. But the channel is non-selective, so Na^+^ and K^+^ counteract each other’s associated water flux. Indeed, in Biedermann et al. [100], appended video simulations do not reveal directional water movement through the α-amino-3-hydroxy-5-methyl-4-isoxazoleproprionic acid (AMPA) receptor channel. Likewise, neuronal swelling in response to NMDA receptor activation involves the cotransporters NKCC1 and swelling-activated KCC2 [101]. Directional water moving as hydration shells of ions conducted through glutamate receptor-activated channels has not been reported. This all supports the argument that water movement through the Na^+^ and K^+^ channels (i.e., Those channels most involved in cationic movement during neuronal activation) are not major conduits of transmembrane water.

## Transport (Carrier) Proteins and Neuronal Swelling

The regulation of water movement across neuronal plasma membranes depends on various transporters which normally cotransport water with their ion substrates. This facilitated transport indirectly sources energy from Na^+^/K^+^ pump cation gradients, so it is not surprising that such transporters are disrupted by ischemia, leading to neuronal swelling.

### Chloride Ion Transporters and Post-SD Neuronal Swelling

**Fig. 10:**
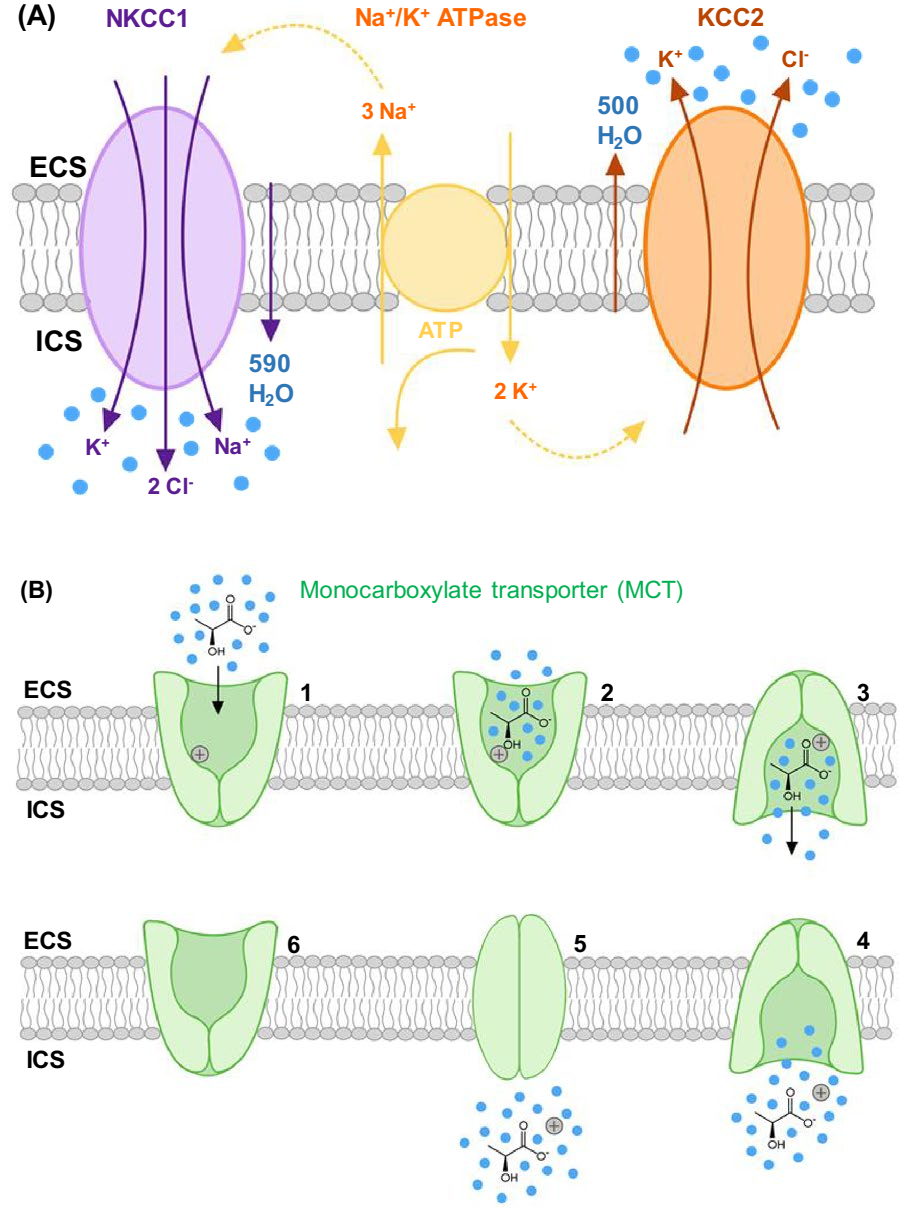
**a** Transmembrane movement of ions and water (blue dots) by the cotransporters NKCC1 and KCC2. Both use energy generated by the Na^+^/K^+^ pump. NKCC1 transports an inflow of 1K^+^, 1Na^+^, 2Cl^−^ with an estimated influx of 590 water molecules. KCC2 transports 1K^+^ and 1Cl^−^ outward with an estimated efflux of 500 water molecules. NKCC1 predominates in early development while KCC2 is the main Cl^−^ extruder (with water) in mature neurons. Figure by J. A. Hellas adapted from Chamma et al. [106]. **b** Schematic of the transport cycle of the proton-linked MCT, an example being L-lactate. The general translocation cycle shows the crypt-like action of a transporter moving water into a neuron. **a1** The transporter is in an outward-open conformation (facing the ECS) where initial proton binding induces (**a2**) L-lactate binding along with water (blue dots). **a3** The transporter changes to an inward-open conformation exposing its contents to the cytosol (into the ICS). **a4** The proton is released into the cell, followed by L-lactate and water. **a5, 6** The transporter returns to an outward-open conformation, the rate-limiting step in the cycle. Figure by J. A. Hellas interpreted from Halestrap’s description of MCT transport [107]. ATPase, adenosine triphosphatase, ATP, adenosine triphosphate, ECS, extracellular space, ICS, intracellular space, MCT, monocarboxylate transporter.

Ion channels passively conduct ions. To maintain selectivity, ions are partially or entirely stripped of their water shells prior to channel entry. However, with cotransporters, water ‘rides along’ with substrates. Mammalian neurons express chloride cation cotransporters (CCCs), specifically Na^+^/K^+^/Cl^−^ (NKCC1) and K^+^/Cl^−^ (KCC2) transporters [62]. Both rely on cation gradients generated by the Na^+^/K^+^ pump as an indirect energy source [102] (Fig. 10A). NKCC1 couples the intracellular transport of two Cl^−^ with one K^+^ and one Na^+^ [102]. KCC2 uses the outward K^+^ gradient to couple efflux of one Cl^−^ and one K^+^. CCCs provide a pathway for transmembrane water transport, even against a prevailing osmotic gradient [62]. Of importance to neuronal swelling, NKCC1 can, along with Cl^−^, facilitate water influx against opposing gradients up to 50 mOsm [62, 103]. This finding was supported by Zhang et al. [102] using cryo-electron microscopy structural analysis of the NKCC1 inward-open state, which contains a continuous pathway for water [102]. Neurons can exhibit volume increases and decreases through NKCC1 and KCC2 transport respectively [58], the latter showing more expression in mature neurons [58]. Inhibiting CCCs and reducing [Cl^−^]_o_ in slices significantly reduces SD-induced dendritic beading [31], indicating the importance of Cl^−^ gradients and altered CCC activity in neuronal swelling. Over hours post TBI, the neuronal NKCC1:KCC2 expression ratio increases [58, 104, 105] and is associated with elevated [Cl^−^]_i_ [58].

Although these CCCs are involved with neuronal swelling, they lack sufficient water-carrying capacity alone to entirely mediate Cl^−^/water influx [102]. Considering the importance of Cl^−^ gradients to neuronal swelling, these CCCs are likely one of several Cl^−^ transporters responsible for water influx. For instance, anion exchange protein 3 (AE3) facilitates Cl^−^/HCO_3_^−^ exchange and is another (less well understood) potential mediator of Cl^-^/water movement [31, 108].

### Monocarboxylate Transporters (MCTs)

Monocarboxylate transporters (MCTs) also move water across membranes by facilitating proton-linked short-chain monocarboxylate (lactate, pyruvate, ketone body) transport [62, 107, 109]. There are four isoforms; MCT1 in BBB endothelium, MCT1 and 4 in astrocytes [110], and MCT2 in astrocytic endfeet and neurons [110, 111]. MCT2 displays a tenfold higher substrate affinity [107, 110]. MCT transport direction depends on the prevailing substrate and proton gradients [107]. The translocation cycle of MCT (Fig. 10b) when transporting L-lactate, involves one proton followed by lactate binding the outward-open conformation. A conformational change to an inward-open state releases substrates and associated water intracellularly.

Because glucose is the brain’s primary energy source, the BBB is relatively lactate-impermeable [110]. However, metabolism altered by hypoglycemia [111], ischemia, or TBI [112, 113] increases BBB monocarboxylate permeability [107] through enhanced MCT1 action. From this, there is MCT1- and MCT4-mediated astrocytic lactate uptake [114], and additional lactate production from glycogen stores to spare residual glucose for neurons [114, 115]. Astrocytic lactate is released into the ECS [112, 113, 116] for neuronal uptake via MCT2 [117]. This is thought to allow for neuronal ATP generation though the lactic acid cycle involving lactate conversion to pyruvate [114, 115]. This provides support for the debated astrocyte neuron lactate shuttle hypothesis.

MCT2 lacks passive water permeability [62], but can cotransport water with its substrate (~ 500 water/lactate) and is enhanced during ischemia [62]. Although MCT2 has potential to induce dendritic water accumulation with ischemia [62, 114], and combined inhibition of AE3 and MCT2 in vivo [31] yielded reduced post-SD beading, specific MCT2 inhibition with 4-CIN [31, 117] does not affect dendritic beading. The role of MCT2 these studies appears to lie in neuronal recovery, aiding in both neuronal water efflux [31] (“Neuronal Recovery and Water Loss” section) and metabolic substrate uptake for functional recovery [117].

### Glucose Transporter (GLUT3)

Glucose is an essential cerebral energy substrate under normal metabolic conditions [118–121]. Neuronal glucose transporter (GLUT3) facilitates glucose influx [119] and its expression is transiently and globally enhanced under ischemia [118, 120]. Its water cotransport properties as observed in *Xenopus* oocytes mimic glial isoform GLUT1 [122], meaning GLUT3 may cotransport ~ 330 water/glucose, in an analogous translocation cycle outlined in Fig. 10A [62]. Given its ischemic upregulation and water cotransport capacity, GLUT3 could mediate neuronal swelling [120, 122, 123]. The caveat is that glucose supply will drop during ischemia, although not entirely [124]. Given the graded transition of reduced cerebral blood flow radiating from the ischemic core, there may be enough residual glucose, particularly in distant regions, to briefly facilitate GLUT3-mediated water uptake [124, 125]. During ischemia, neurons are metabolically-stressed, leading to brief enhancement of GLUT3 glucose and water uptake, causing the accumulation and trapping of this water intracellularly (“*N*-Acetyl-L-Aspartate/*N-*Acetylaspartylglutamate Metabolism and Molecular Water Pump Cycle” section). This process has potential to contribute to neuronal swelling and may help keep the metabolically-stressed neurons alive for a short time following ischemic onset. However, the exact magnitude and duration of GLUT3-mediated water intake have not been investigated.

### Metabolic Water

As mentioned, briefly-enhanced neuronal glucose and water uptake is possible through GLUT3. We propose that this process further swells neurons via increased oxidative glucose metabolism, causing the production and trapping of metabolic water. Because enhanced GLUT3 activity under conditions such as prolonged stimulation [126] corresponds to elevated metabolic water production [127], the brief period of enhanced GLUT3 activity as described above ("Glucose Transporter (GLUT3)" section) may also enhance intraneuronal metabolic water production and trapping, contributing to swelling. Similar to GLUT3 activity, this is likely brief, lasting only until circulating oxygen is entirely lost. Although cerebral blood flow and oxygen extraction fraction poststroke have been observed, each showing graded reductions moving away from the ischemic core [124, 125, 127], the exact duration and contribution of metabolic water production to neuronal swelling are unknown.

### *N*-Acetyl-L-Aspartate/*N*-Acetylaspartylglutamate Metabolism and Molecular Water Pump (MWP) Cycle

**Fig. 11:**
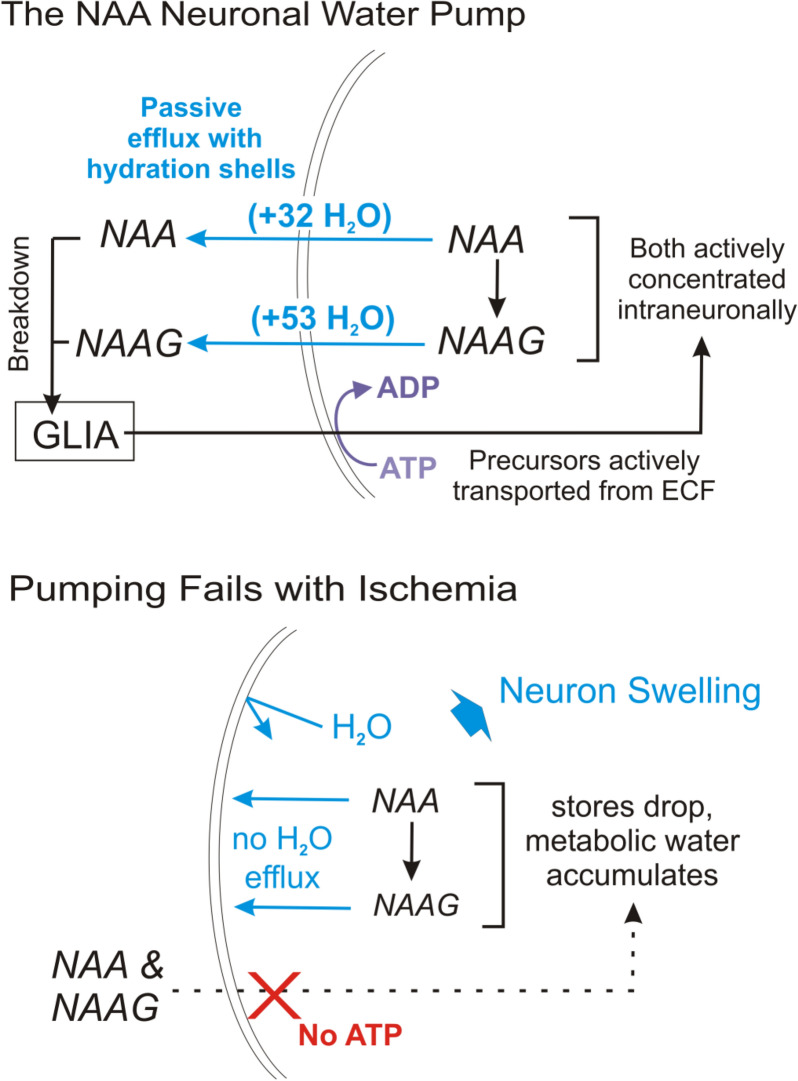
A hypothesized neuronal water pump involving NAA and NAAG, a derivative of NAA and L-glutamate. Adapted from Baslow [131]. **a** Possible mechanism of transmembrane neuronal water transport under normal conditions. Water is also cotransported with NAA and NAAG via cyclic hydrolysis and resynthesis. **b** Proposed change in water pumping under lost ATP production during ischemia. NAA and NAAG-mediated water efflux is inhibited, resulting in neuronal swelling. Figure by R. D. Andrew. ADP, adenosine diphosphate, ATP, adenosine triphosphate, ECF, extracellular fluid, NAA, *N*-acetylaspartate, NAAG, *N*-acetylaspartylglutamate.

*N*-acetyl-L-aspartate (NAA) is a derivative of aspartic acid synthesized and stored in neurons [127–129]. NAA synthesis is cyclic, thought to be associated with a transmembrane molecular water pump (MWP) system [127]. Normally, following synthesis, NAA leaves the neuron down its gradient, into the ECS (Fig. 11a) [127]. *N*-acetylaspartylglutamate (NAAG) is analogous, although less common, but follows the same cycle. Oligodendrocytes [127] and possibly astrocytes [130] take up NAA/NAAG for their hydrolysis, and the by-products are sent back to neurons for resynthesis [127, 128]. NAA/NAAG efflux is associated with the total efflux of ~ 85 metabolic water molecules [127, 128]. Assuming that most CNS NAA/NAAG production is neuronal, this system can theoretically remove up to 50% of neuronal metabolic water produced under normal conditions. With ischemia, NAA/NAAG synthesis is reduced [127], yet the rate of NAA/NAAG hydrolysis remains the same. This destroys the NAA/NAAG gradients (Fig. 11b) [127]. We hypothesize that metabolic water accumulates, promoting acute neuronal swelling during ischemia.

### Neuronal Recovery and Water Loss

The functional threshold of decreased perfusion with ischemia is the minimum perfusion allowing neuronal function without irreversible damage [132]. The functional threshold of a cell during ischemia depends on cell type, region, infarct time, and magnitude [132, 133]. Generally, total brain death does not occur immediately following acute ischemia provided there is timely reperfusion [125], and there is a 3 to 6 hour window of opportunity post-insult where some brain tissue can be salvaged [29, 132]. During reperfusion and recovery, neurons must remove excess intracellular water [29, 132]. Similar to water accumulation, the mechanisms underlying neuronal water efflux with ischemic recovery are poorly-characterized.

#### Water Efflux via Chloride Cotransporters

As mentioned, the expression ratio of neuronal NKCC1:KCC2 increases post-TBI [58, 104, 105] ("Chloride Ion Transporters and Post-SD Neuronal Swelling" section) and is partly responsible for elevated [Cl^−^]_i_ [58, 134]. Although return to normal NKCC1:KCC2 expression, particularly normal NKCC1 efflux function, has potential to aid in the removal of excess water from swollen neurons, altered KCC2 expression is observed up to 1 week post-SD from TBI [58]. It is unlikely that return to normal chloride cotransporter expression would aid in neuronal recovery immediately post SD. It is possible however, that expression of these transporters is modulated by a slower mechanism such as transcriptional regulation [134]. More investigation is required regarding NKCC1 expression and its rate of recovery post-SD.

#### MCT Reversal

As mentioned, MCT2 reversibly transports lactate (or pyruvate) and water depending on prevailing gradients [62, 107, 109–111, 113]. Selective MCT2 inhibition with 4-CIN [31] in slices indicated maintenance of dendritic beading after ~ 4 minutes of normoxic SD, while slices with functional MCT2 returned to near-control beading. Thus, with MCT2 upregulation during and following SD [107], it is possible that a reversed MCT2 removes lactate and water, mediating neuronal recovery water post-ischemia.

#### NAA/NAAG MWP Restoration

Once appropriate metabolic conditions are met with reperfusion [127, 128, 135], neurons can resume NAA/NAAG synthesis ("N-Acetyl-L-Aspartate/N-Acetylaspartylglutamate Metabolism and Molecular Water Pump (MWP) Cycle" section), restoring NAA/NAAG gradients and water pumping (Fig. 11) as proposed by Baslow et al. [127]. Restoration of MWP function following brief ischemia would effective for neuronal water efflux given its high transport capacity [128]. However, this pump still requires molecular characterization.

## Conclusions

In cases of brain ischemia, acute cytotoxic edema is the early and common aspect of neurological damage [5]. It is a severe clinical problem yet is poorly-characterized due to the difficulty in monitoring microscopically in situ. Studies of brain cell swelling in the 1980s and 1990s focused on visualizing astrocytic and neuronal volume changes in isolated cells. Such studies demonstrated volume changes and RVD/RVI, usually in response to unphysiological osmotic stress. Studies likewise examined the effects of simulated ischemia on isolated or cultured neurons, which do not generate SD. These preparations were far from ideal, lacking physiological neuron-glia relationships, a restricted ECS, synaptic interactions, and intrinsic membrane conductances. Early in the new millennium, real-time imaging using 2-PLSM enabled brain cell volume to be monitored in live brain slices and in situ under osmotic or ischemia-like stress. It became apparent that astrocytes are osmoresponsive while neurons are not (except for specific osmosensitive neurons). Both cell types dramatically swell when Na^+^/K^+^ pump compromise causes SD. Cortical neurons in swollen, postischemic gray matter demonstrate reduced excitability for hours to days, in contrast to osmotically-swollen tissue where excitability increases over minutes. Transmembrane ion gradients, established primarily by the Na^+^/K^+^ pump, indirectly drive ion/water cotransporters and molecular water system pumping. Specifically, altered function and water cotransport capacity via Cl^−^ transporters, MCT2, and GLUT3 are thought to contribute to neuronal swelling, but the specific molecular details of this dysregulation require much more research. Further, altered neuronal metabolism may induce NAA/NAAG MWP malfunction (Fig. 11). The question of which transporters are reversed or upregulated to salvage swollen neurons post-SD also requires investigation. Of additional interest should be intraneuronal metabolic water production and trapping, as it is likely a significant mediator of swelling, but is not well studied. A better understanding of the molecular mechanisms underlying SD and the neuronal swelling that results will help treat neurological damage arising acutely following ischemia.

## Appendix 1: Regulatory Volume Decreases or Increases (RVD or RVI) by Neurons

Almost all brain slice studies to date do not support either RVD/RVI activation over a period of minutes in nonosmosensitive neurons [3, 13]. This is surprising because many isolated cell studies over 30 years have documented both RVD and RVI. However, most of these studies used extreme hypoosmotic conditions, causing swelling far beyond what could be tolerated in vivo given the confining skull and dura [3, 38]. One brain slice study did document RVD [43] but used strongly hypoosmotic shifts [136], so physiological relevance becomes a concern.

Live brain slice studies hold more promise than isolated cell studies because extreme swelling which would otherwise be fatal in situ can be monitored while maintaining local neuron structure, local synaptic connectivity, intimate neuron-glial relationship, and the extracellular matrix [137]. Although acutely-dissociated or cultured cells also allow for extreme swelling from lacking the normally confined ECS and synaptic connectivity [138], the isolation process causes neurons to lose surrounding structural elements including most of their dendritic arbor [139]. Isolated neuron preparation also necessitates exposure to solutions with different osmolyte concentration, contents, and temperature compared to typical physiological conditions. Finally, cells may be sourced from embryos or from cell lines with properties that develop differently in the dish than adult neurons, causing differences in physiological properties which may include receptor expression or neurotransmitter release, among others [140]. Examining volume change only in isolated cells thus requires a number of assumptions: (1) That the applied aCSF osmolality shift does not evoke a novel response due to isolation techniques; (2) That volume changes observed in cell bodies are representative of their more distal (and far more extensive) processes [3]; (3) That any osmotic responses exclude effects upon chemical synapses [3, 13, 15, 28]. Therefore, evoked changes to brain cell volume are better examined in brain slices preferably using 2-PLSM or in situ.

### No Obvious Osmotic RVD by Astrocytes

Astrocytes swell rapidly under many pathological conditions, including acute hypoosmotic stress [2, 6, 141], so glia are an important contributor to the osmotic cerebral volume changes [7, 142]. In brain slice studies using 2-PLSM, there is no evidence that astrocytes exhibit RVD over minutes of physiological hypoosmotic stress. In support, LT change shows an inverse linear relationship with the degree of shift in osmolality; this remains stable over many minutes (Fig. 4) [3, 7, 49]. Oligodendrocytes also demonstrate passive swelling under acute hypoosmotic stress similarly to astrocytes, as modeled and observed by Vargovà et al. [143]. Cell volume changes were measured indirectly by monitoring the ECS volume shifts in rat brain and spinal cord slices upon application of hypoosmotic and hyperosmotic aCSF.

### No Obvious Osmotic RVD/RVI by Regular (i.e., Nonosmosensory) Neurons

Although there is strong evidence that the brain can adapt to subacute (hours and days) osmotic stress [35], there is little support from brain slice studies that neurons or astrocytes display RVD/RVI upon rapid, physiologically relevant osmotic stress [2, 3, 7]. For example, some neuronal precursor cells display RVD with hypoosmolality at or below − 30% which is ~ 90 mOsm below normal plasma osmolality [16] (normally maintained between 280 and 295 mOsm [144]). These conditions are well below any reasonable physiological, or pathophysiological plasma osmolality, even in patients with severe hyponatremia where plasma osmolality drops between 236 and 244 mOsm [145]. Osmolality this low results in severe symptoms including seizure. For experimental osmolality reductions by 15% (up to ~  − 45 mOsm), there is no evidence of RVD in neurons [2, 3, 16]. Even under pathophysiological hypoosmotic stress (~ − 15%), neuronal cell bodies, dendrites, and axons, are resistant to both osmotic swelling and shrinkage [2, 16]. So, unlike many studies of cultured cells derived from neurons, there is little experimental evidence in slices that cortical neurons undergo detectable volume changes in response to pathophysiological osmotic stress, much less RVD or RVI over seconds or minutes.
